# Pyroptosis-related gene-based prognostic signature for predicting the overall survival of oral squamous cell carcinoma patients

**DOI:** 10.3389/fsurg.2022.903271

**Published:** 2022-08-19

**Authors:** Deliang Zeng, Xiao Wang, Shuning Zhang, Ao Zheng, Qingfeng Huang, Lingyan Cao

**Affiliations:** Department of Prosthodontics, Shanghai Engineering Research Center of Advanced Dental Technology and Materials, Shanghai Key Laboratory of Stomatology and Shanghai Research Institute of Stomatology, National Clinical Research Center for Oral Diseases, Shanghai Ninth People’s Hospital, College of Stomatology, Shanghai Jiao Tong University School of Medicine, Shanghai, China

**Keywords:** oral squamous cell carcinoma, pyroptosis, immune infiltration, overall survival, prognostic signature

## Abstract

**Purpose:**

Oral squamous cell carcinoma (OSCC) is the most common oral cancer worldwide. Pyroptosis is a type of programmed cell death mediated by caspase, accompanied by an inflammatory response, and plays an important role in cancer progression. The purpose of this study was to explore and identify potential biomarkers and further elucidate the potential role of cell pyroptosis in OSCC.

**Methods:**

We regarded the samples from The Cancer Genome Atlas database as a training dataset, screened differentially expressed genes (DEGs), and further screened out OSCC phenotypic characteristic genes by using weighted gene co-expression network analysis. The analysis of 42 known pyroptosis-related genes showed that Psuch genes were widely expressed, mutated, and methylated in OSCC samples.

**Results:**

Through correlation analysis, we identified our OSCC pyroptosis-related DEGs. To further evaluate the prognostic value of pyroptosis-related regulators, we constructed a seven gene-based prognostic signature using Cox univariate analysis and least absolute shrinkage and selection operator Cox regression analysis. Meanwhile, we found that patients in the low-risk group had higher immune infiltration. Moreover, our results also indicated significant differences in sensitivity to cisplatin and gefitinib between the high-risk and low-risk groups.

**Conclusion:**

Our study successfully constructed the pyroptosis-related prognostic signature, which might play a potential prediction role in OSCC prognosis. Our findings also suggested that pyroptosis-related regulators might be novel biomarkers for tumor diagnosis and treatment in OSCC.

## Introduction

Oral squamous cell carcinoma (OSCC) is a common malignant tumor type of oral cancer and brings huge health burden to society (1). According to the report of the World Health Organization (WHO), the annual incidence rate of OSCC worldwide is more than 300,000 cases globally (2). The prognosis of OSCC patients is poor, with an approximately 50% 5-year survival rate due to the limited understanding of molecular mechanisms (3). In the last few decades, although the technological and biological advances recently have been developed, the prognosis of this disease has not changed (4). Therefore, the identification of novel and efficient prognostic predictors is necessary and may guide personalized clinical treatment.

Pyroptosis is a programmed mode of cell death in the body's immune response, mediated by cysteine aspartate-specific proteases (caspase), accompanied by an inflammatory response, and has been reported to play an important role in cancer development (5, 6). Pyroptosis is thought to be a response to infection and is reported to be usually triggered by inflammasome (7). Increasing evidence revealed that pyroptosis could affect the overall survival of cancer patients by changing the immune infiltration levels in the tumor microenvironment (TME) (8). For example, Liang et al. constructed a prognostic model using pyroptosis-related genes and found that this model might efficiently predict the prognosis of gastric cancer patients (9). Cao et al. identified a series of long non-coding RNAs regulating the progression of pyroptosis and then established a pyroptosis-related lncRNA prognostic model for ovarian cancer (10). In addition, many reports confirmed that the pyroptosis level was significantly correlated to immunotherapy response in diverse types of human cancers such as glioma (11), pancreatic adenocarcinoma (12), and esophageal adenocarcinoma (13). These studies further determined the crucial value of pyroptosis in cancer, as well as the patient prognosis. However, the potential prognostic significance of pyroptosis in OSCC is still lacking and needs to be extended for guiding clinical directed treatment.

In the present study, we identified 278 potential regulators that are differentially expressed in OSCC, which are significantly associated with 42 pyroptosis genes by using the *limma* and weighted gene co-expression network analysis (WGCNA) function packages in R. The least absolute shrinkage and selection operator (LASSO) Cox regression model is a popular algorithm widely used in medical research for feature selection (14–17). Further, we integrated the LASSO Cox regression model analysis to narrow down the range of candidate genes and finally screened out seven pyroptosis-related regulators to construct a prognostic model. Seven pyroptosis-related regulators determined the efficiency and accuracy of the predictive model in both datasets, including training and validation sets.

Moreover, we also evaluated the immune infiltration levels between two risk groups and found that patients in the low-risk group had a stronger infiltration ratio of the microenvironment. Our results suggested that the prognostic model might guide the use of antineoplastic drugs such as gefitinib and cisplatin clinically.

## Materials and methods

### Data collection and processing

The RNA FPKM expression profiles and clinical information of the training dataset (316 OSCC samples and 30 normal samples) were downloaded from the HNSCC cohort of The Cancer Genome Atlas (TCGA-HNSC). Meanwhile, similar information about the validation dataset (40 OSCC samples) was generated from the International Cancer Genome Consortium (ICGC). The original data is normalized by the log_2_^(*x*+1)^ method, and the standardization was performed based on the robust multi-array method. Subsequently, the differential expression analysis was carried out by the *limma* function package of R language (18), with |log 2 [fold change (FC)]| > 1 and *P* ≤ 0.05 as the set.

### Weighted gene co-expression network analysis

The “WGCNA” function package of R language was used to perform the WGCNA as previously described (19). In brief, according to the gene expression value of genes, genes with high similarity were divided into the same module by the dynamic clipping tree method, and the module was identified. Next, the module eigengene (ME) value and the correlation coefficient between the ME value and phenotype of interest were calculated. Furthermore, phenotypes referred to disease states, specifically, under the significant correlation coefficient (*P* < 0.05); the larger the absolute value of ME, the closer the module to the benefit phenotype.

### Gene Ontology and Kyoto Encyclopedia of Genes and Genomes pathway enrichment analysis

The 42 pyroptosis-related genes were obtained from previous studies and the GO:0070269 pathway (20–24), and they are presented in **Supplementary Table S1**. For 42 genes, Gene Ontology (GO) analysis (including biological process, molecular function, and cellular component) and Kyoto Encyclopedia of Genes and Genomes (KEGG) pathway enrichment analysis was conducted by the clusterProfiler function package of R language (25), with *P* < 0.05 as the significant threshold.

### Construction and validation of the prognostic signature

To screen out the key genes that associated with OSCC prognosis, univariate Cox regression analysis was applied by using the “survival” function of R language. Then, a prognosis model was constructed with these candidate genes. Risk score = Gene 1 * β1 + Gene 2 * β2 +… Gene n * βn. The β symbol represents the regression coefficient for each gene of interest obtained from the training dataset. Subsequently, we divided patients into high- and low-risk groups based on the median risk score. The prognostic effect was evaluated by using the time-dependent ROC curve using the “*timeROC*” function. In addition, the validation dataset was used to verify the accuracy of this prognostic signature.

### Immune infiltration analysis

XCELL in TIMER (http://timer.comp-genomics.org/) was used to evaluate the percentage of immune cell types, that is, the infiltration levels of diverse types of immune cells among OSCC samples as previously described (26). Further, the differences in immune cell invasion between two risk groups was assessed using SSGESA according to the previous studies (27).

### Evaluation of the correlation between risk score and clinical response to chemotherapeutic agents

To investigate whether patients with OSCC could benefit clinically from immunotherapy, chemotherapy combined with immunotherapy has been shown to be more effective than either alone. We investigated the correlation between risk score and clinical response to chemotherapeutic agents. *pRRophetic* is an R software package designed to assess clinical drug responses (the half-maximal inhibitory concentration, IC50) by integrating baseline gene expression levels and drug sensitivity data in cancer cell lines (1, 28).

### Statistical analysis

R V4.1.0 (http://www.Rproject.org) was used to perform the statistical evaluation. The Wilcoxon test was used to compare the differences between the two groups. *P* < 0.05 was considered the significant threshold.

## Results

### Identification of phenotypic-specific differentially expressed genes

**Figure 1:**
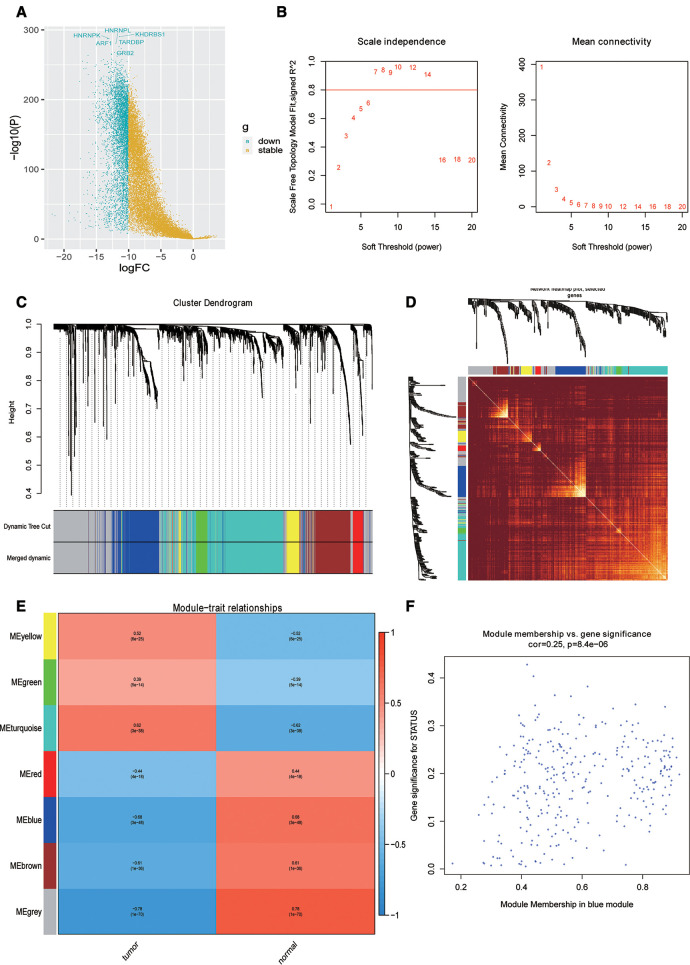
Identification of phenotypic-specific differentially expressed genes (DEGs). (**A**) Differential gene volcano map between Oral squamous cell carcinoma (OSCC) samples and normal samples. (**B**) Soft threshold power analysis for generating the scale-free fitting index of network topology. (**C**) Hierarchical cluster analysis to detect co-expression clusters with corresponding color assignments. (**D**) Heat map depicting the topological overlap matrix between DEGs based on co-representation module (TOM). (**E**) Heat maps of correlations between modular signature genes and OSCC phenotypes. (**F**) Scatter plot of the correlation between blue module and genes most correlated to phenotype.

First, the differential expression analysis between OSCC samples and normal samples was performed, and we identified 2,956 differentially expressed genes (DEGs) (Figure 1A). To perform the WGCNA analysis, the soft threshold power analysis was used to obtain the scale-free fitting index of network topology (Figure 1B). Here, according to the WGCNA algorithm, the gene expression network was assumed to obey the scale-free distribution, and the gene co-expression network was constructed. Subsequently, we constructed the hierarchical clustering trees by calculating the dissimilarity coefficients of different nodes. In addition, we grouped high-similarity genes into the same module and low-similarity genes into different modules and visualized these modules. For WGCNA analysis of 2,956 DEGs, we set the soft threshold to 7 to construct a scale-free network (Figure 1C). Further, the adjacency matrix and topological overlap matrix were constructed and are shown in Figure 1D. Finally, seven modules were obtained based on average hierarchical clustering and dynamic tree clipping (Figure 1E). The MEs and Pearson correlation coefficients for disease status were calculated for all modules to determine which module was associated with OSCC, and we found that the blue module (*R* = 0.68, *P* = 3e-48) was significantly negatively correlated to disease status (Figure 1E). Scatter plot of correlation between the blue module most related to phenotype and genes is shown in Figure 1F. Hence, the 310 genes in the blue module were selected for the subsequent analysis.

### Evaluation of pyroptosis genes in oral squamous cell carcinoma (OSCC)

**Figure 2:**
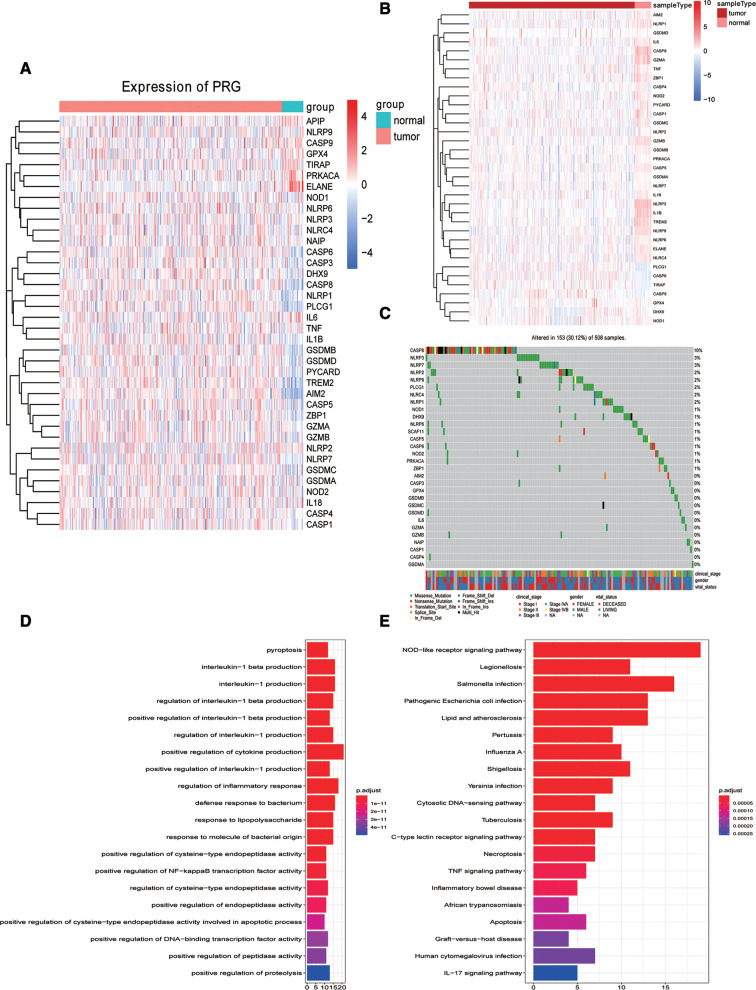
Evaluation of pyroptosis genes in Oral squamous cell carcinoma. Expression heat map (**A**), methylation heat map (**B**), and top 30 mutant waterfall diagrams (**C**) of 42 pyroptosis-related genes in the training dataset. Gene Ontology analysis (**D**) and Kyoto Encyclopedia of Genes and Genomes pathway enrichment analysis (**E**) using 42 pyroptosis-related genes in the training dataset.

To assess the change of pyroptosis genes in OSCC, we downloaded the 42 pyroptosis-related genes from previous studies and the GO:0070269 pathway. As shown in Figures 2A,B, we found that the expression and methylation modification of 42 pyroptosis-related genes were significantly changed in OSCC samples compared to normal samples. Meanwhile, we observed the extensive mutations of pyroptosis-related genes in OSCC samples compared to that in normal samples (Figure 2C). Then, GO and KEGG enrichment analyses were carried out using these 42 pyroptosis-related genes, and the results revealed that these genes were mainly enriched in pyroptosis, interleukin-1 β production, interleukin-1 production, regulation of interleukin-1 β production, and other biological processes (Figure 2D) and also in the NOD-like receptor signaling pathway, legionellosis, *Salmonella* infection, pathogenic *Escherichia coli* infection, and other signaling pathways (Figure 2E).

### Identification of pyroptosis-related key regulators

**Figure 3:**
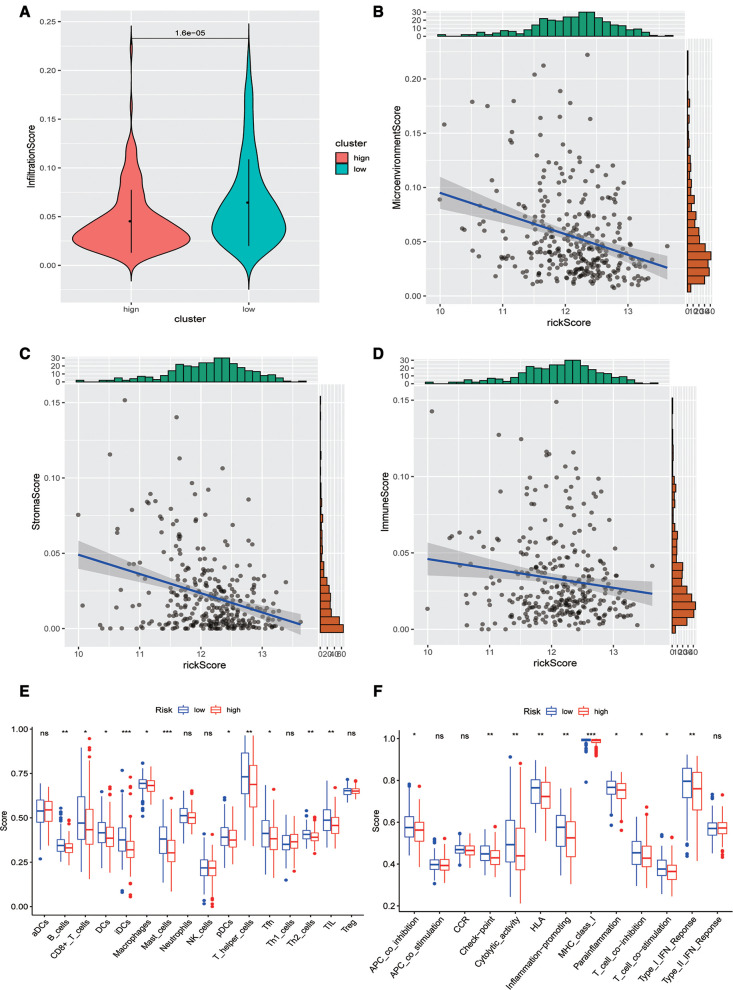
Identification of pyroptosis-related key regulators. (**A**) Correlation network diagram of 278 pyroptosis-related key regulators and 42 pyroptosis-related genes. Expression heat map (**B**,**C**) and methylation heat map (**D**) of 278 pyroptosis-related key regulators in the training dataset. (**E**) PCA clustering of 278 pyroptosis-related key regulators in the training dataset. (**F**) Prognostic forest map of 13 pyroptosis-related signatures.

To explore which key genes might affect the process of pyroptosis and thus the occurrence and development of OSCC, we conducted the correlation analysis of 310 DEGs in the blue module and 42 pyroptosis-related genes and obtained a total of 278 key genes that might impact the pyroptosis-related genes (Figure 3A), suggesting that these genes might regulate the pyroapoptotic process of OSCC. The expression and methylation modification of 278 pyroptosis-related regulators were significantly changed in OSCS samples compared to those in normal samples (Figures 3B,D). PCA analysis showed that these 278 pyroptosis-related regulators could distinguish OSCC samples from normal samples (Figure 3C). Finally, by performing the Univariate Cox regression analysis combined with clinical information of patients, we screened out 13 key pyroptosis-related key regulators that are closely correlated to OSCC prognosis (Figure 3E). These genes were *NFKBIL2*, *FAM72D*, *FAM72B*, *COL27A1*, *TAF1A*, *AURKAPS1*, *DDX12*, *CKS2*, *HIST1H3F*, *MAML3*, *LOC283314*, *FST*, and *MCEE*.

### Construction and validation of the prognostic model

**Figure 4:**
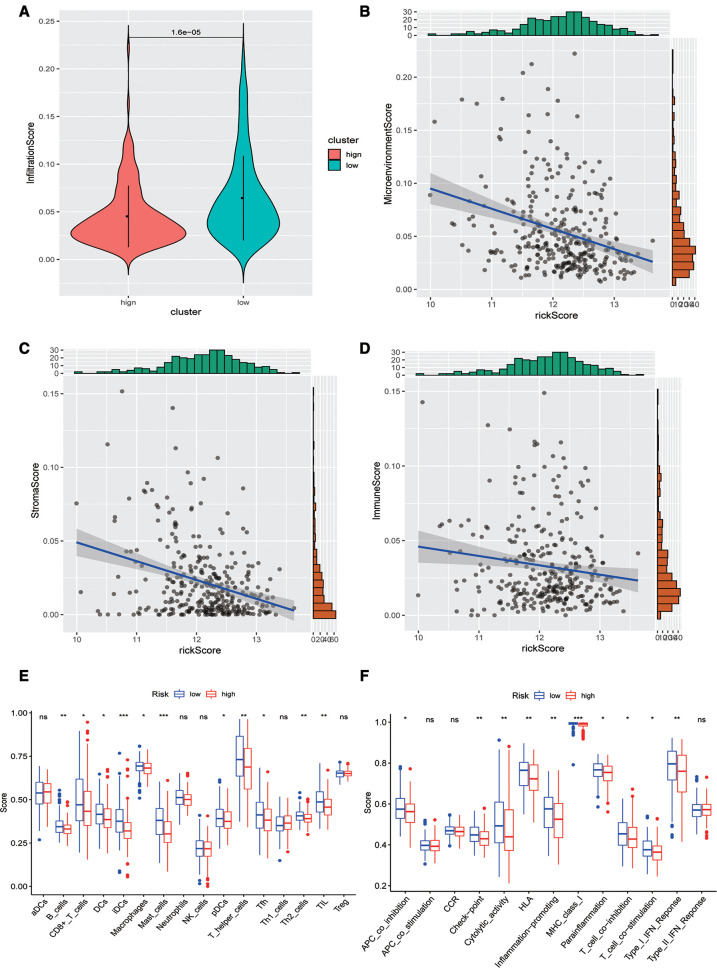
Construction and validation of the prognostic model. (**A**) Correlation network diagram of 13 pyroptosis-related key regulators. (**B**) Parameter selection in least absolute shrinkage and selection operator (LASSO) regression. (**C**) Coefficient distribution in LASSO regression. Survival analysis based on risk scores in the training set (**D**) and validation dataset (**D**). (**E**) Univariate Cox risk regression considering other clinical factors in the training dataset. (**F**) Univariate Cox risk regression considering other clinical factors in the validation dataset.

Then, we evaluated the prognostic values of these pyroptosis-related key regulators. Correlation analysis showed that there was an obvious expression correlation among the 13 pyroptosis-related key regulators (Figure 4A). Then, the penalty coefficient of LASSO regression was generated by using the *glmnet* function of R language, and we found that as lambda increases, the degrees of freedom and residuals decrease (Figure 4B). Genes with penalty factor 0 were reserved as the final screening variables (Figure 4C). Therefore, seven genes (*FAM72D*, *COL27A1*, *HIST1H3F*, *MAML3*, *LOC283314*, *FST*, and *MCEE*) were retained, and their corresponding penalty coefficients are given in Table 1. Subsequently, risk scores were the sum of the product of the penalty coefficient and expression value. Then, we set the median value of risk scores as the threshold and separated patients in the training dataset into high- and low-score groups; survival analysis revealed that patients in low-score groups had a better prognosis than that in high-score group (Figure 4D). Moreover, a similar method was applied to the validation dataset, and the same trend was observed (Figure 4E). Based on the clinical characteristics of univariate Cox risk regression combined with the training set and validation set, the results demonstrated that the risk score was a relative independent prognostic indicator considering the age, gender, grade, and stage in both the training dataset (Figure 4F) and validation dataset (Figure 4G).

**Table 1:** Corresponding penalty coefficients of seven retained signatures.

Gene	Coefficients
*FAM72D*	1.2739586562672
*COL27A1*	1.91803710252839
*HIST1H3F*	0.410730915853282
*MAML3*	−0.532129522990241
*LOC283314*	0.205946072872406
*FST*	1.35454747894868
*MCEE*	−0.574992503960348

### Analysis of immune infiltration levels

**Figure 5:**
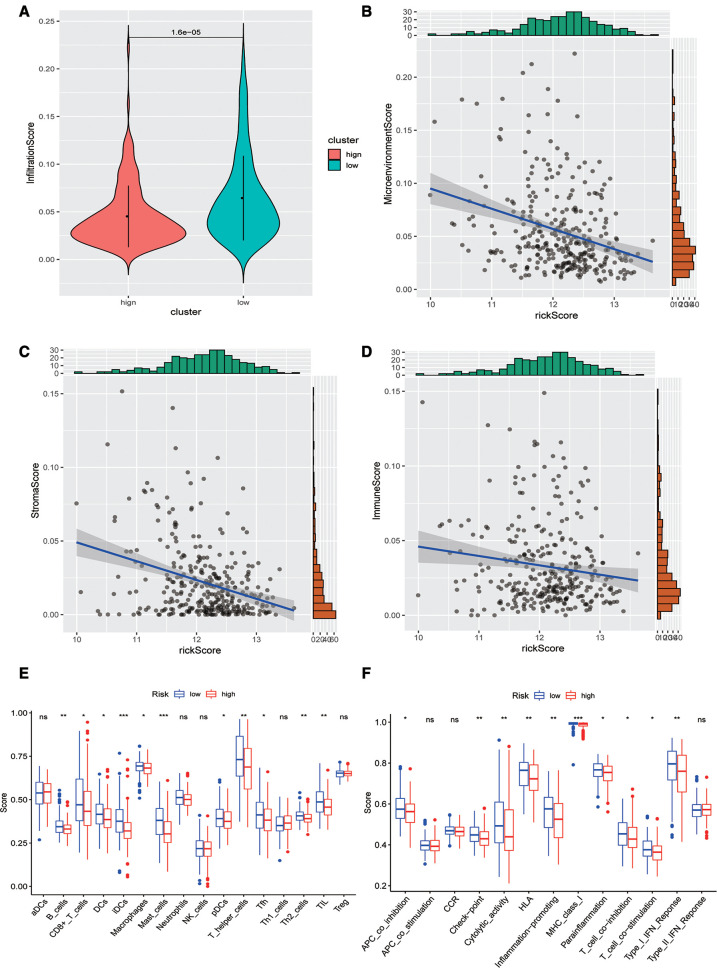
Analysis of immune infiltration levels. (**A**) Comparison of immune microenvironments between high and low-risk groups. (**B**–**D**) Scatter plot of the correlation between total score, stromal cell score, immune cell score, and risk score in the microenvironment. (**E**) Difference between groups at high or low risk of Oral squamous cell carcinoma under different immune cell types. (**F**) Differences between groups at high and low risk of OSCC under different activated immune pathways.

Subsequently, we evaluated the difference in immune infiltration between high- and low-risk score groups and found that the levels of immune infiltration in the high-risk group were obviously lower than those in the low-risk group (Figure 5A). The correlation scatter plot also showed that the infiltration ratio of immune cells and stromal cells in the tumor microenvironment was negatively correlated to the prognostic of OSCC patients determined by our prognostic model. The higher the risk score, the lower the infiltration ratio of the microenvironment (Figures 5B–D). In addition, patients in the low-risk group showed a significant survival advantage due to the abundant infiltration of innate immune cells, including B cells, iDCs, TIL, and activated CD8 T cells (Figure 5E). Meanwhile, check-point, cytolytic_activity, MHC_class_I, T_cell_co-inhibition, and other immune functions exhibited higher levels in the low-risk group (Figure 5F).

### Evaluation of the association between the risk model and chemotherapy response

**Figure 6:**
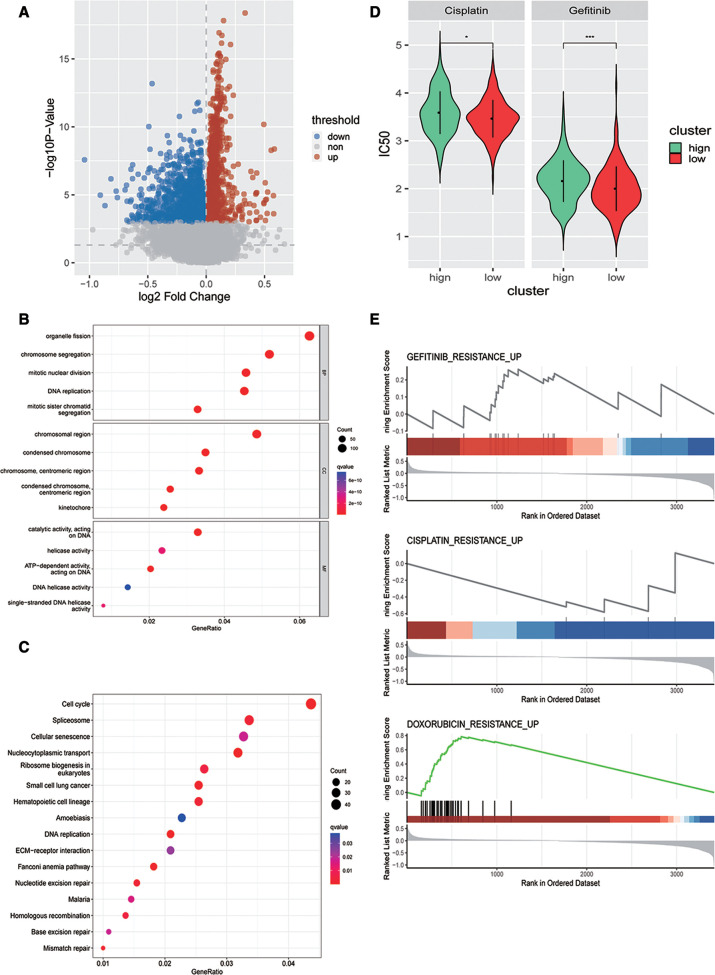
Evaluation of the association between the risk model and chemotherapy response. (**A**) Volcano map of DEGs in high- and low-risk groups in the training dataset. Gene Ontology analysis (**B**) and Kyoto Encyclopedia of Genes and Genomes pathway enrichment analysis (**C**) of DEGs in two risk groups in the training dataset. (**D**) Predicted differences in drug sensitivity between high- and low-risk groups. (**E**) GSEA analysis of differential genes in drug resistance pathways between high- and low-risk groups.

Next, we analyzed the DEGs between high- and low-risk score groups= and identified 2,830 DEGs (Figure 6A). GO analysis revealed that these DEGs were closely enriched in mitotic nuclear division, centromeric region, ATP-dependent activity, acting on DNA biological processes (Figure 6B), and also enriched in DNA replication, ECM−receptor interaction, and mismatch repair signaling pathways (Figure 6C). To investigate whether patients with OSCC could benefit from immunotherapy clinically, we evaluated the risk model and chemotherapy response (cisplatin and gefitinib) using the *pRRophetic* function of R language and found that there were significant differences in sensitivity to cisplatin and gefitinib between high- and low-risk groups (Figure 6D). Finally, we conducted gene set enrichment analysis (GSEA) based on the DEGs between high- and low-risk groups to explore the drug resistance signaling pathway, and the results showed that the DEGs could affect the drug resistance of gefitinib, cisplatin, adriamycin, and other drugs (Figure 6E). These findings suggested that our model might guide the use of antineoplastic drugs clinically.

## Discussion

Oral cancer is becoming a global health problem due to its relatively high incidence and mortality. Also, due to the poor prognosis of OSCC after surgery combined with chemoradiotherapy (29, 30), increasing research studies have paid more attention to the identification of efficient prognostic predictors in OSCC such as microRNA levels (31), long non-coding RNAs (32), and apoptosis-related genes (33). Although the role of pyroptosis in human cancers have been studied, their prognostic values in many other malignancies have not been well characterized. Pyroptosis occurs in pathogen-infected cells as a manifestation of programmed cell death, which induces an inflammatory response in the body (34) and has diverse roles. Pyroptosis has tumor growth inhibitory effects on colorectal and skin cancers (35, 36); hence, we hypothesized that pyroptosis might also play a potential role in OSCC. In this study, our results systematically analyzed the expression of pyroptosis-related genes in OSCC samples and further identified a series of potential genes that regulated these genes. Then, a prognosis model was constructed based on seven optimized pyroptosis-related regulators, and our data determined that the prognostic model might be useful for predicting the prognosis of OSCC patients.

In the present study, seven pyroptosis-related regulatory genes (*FAM72D*, *COL27A1*, *HIST1H3F*, *MAML3*, *LOC283314*, *FST*, and *MCEE*) were finally selected for the construction of a prognostic model, and our data confirmed that the seven gene-based prognostic model could accurately predict the outcome of OSCC patients. The prognostic values of these seven key genes in OSCC or other human diseases have been investigated. For example, a previous study established a signature using nine hepatitis C virus-induced genes including FAM72D, which could better predict the prognosis of hepatic cancer and also provide a novel way to investigate the potential mechanism of HCVIGs in this disease (37). Hu et al. found the downregulation of COL27A1 in poor segmental congenital scoliosis (38), and Laura et al. revealed a steel syndrome patient due to the compound heterozygous COL27A1 mutations in the eye (39). Garciaz et al. found that the downregulation of the HIST1H3F level could predict a better prognosis in patients with acute myeloid leukemia (40). A recent study performed an integrated analysis and revealed that miR-20°c-3p and miR-52°c-3p could intermediate core-regulatory genes including MAML3 to affect stemness and metastasis in gastric cancer (41). Liu et al. demonstrated that the downregulated level of FST was significantly correlated to the poor survival of patients with triple-negative breast cancer (42). Tang et al. analyzed the genetic polymorphisms that affected pancreatic cancer survival and found that MCEE level was closely related to triple-negative breast cancer prognosis (43). Although the prognostic values of these seven pyroptosis-related regulatory genes in human cancers have been partially explored, their significance in OSCC remains unclear. Here, we investigated the prognostic values of the seven genes and revealed that this seven-gene-based prognostic model could be potentially used for clinical prediction of overall survival. Our findings might provide a new way for personalized treatment.

Gefitinib is a tyrosine kinase inhibitor with low molecular weight (44), and cisplatin is a cytotoxic, DNA-damaging alkylated chemotherapy drug (45). Increasing clinical applications have reported that these two drugs were widely used for the treatment of different types of solid tumors including OSCC (46). However, the efficiency of two drugs in tumors often depends on the tumor size, stage, and other clinical factors. Hence, a well understanding of chemotherapy response against different tumor types is more urgent, which might contribute to applying efficient chemotherapy drugs. In this study, there were significant differences in sensitivity to cisplatin and gefitinib between high- and low-risk groups. These findings might provide clinical medicine for OSCC.

In conclusion, we constructed a seven-gene-based prognostic signature based on seven pyroptosis-related regulators and even investigated the correlation between the risk model and chemotherapy response. The results might guide the use of antineoplastic drugs clinically. However, more patient data were needed to be collected for verifying the accuracy of our prognostic model.

## Data Availability

Publicly available datasets were analyzed in this study. This data can be found here: The datasets used and/or analyzed during the current study available from the corresponding author on reasonable request.
